# Virtual Reality as an Embodied Tool to Enhance Episodic Memory in Elderly

**DOI:** 10.3389/fpsyg.2016.01839

**Published:** 2016-11-17

**Authors:** Claudia Repetto, Silvia Serino, Manuela Macedonia, Giuseppe Riva

**Affiliations:** ^1^Catholic University of the Sacred Heart, Department of PsychologyMilan, Italy; ^2^Istituto Auxologico Italiano, Applied Technology for Neuro-Psychology LabMilan, Italy; ^3^Johannes Kepler University, Information EngineeringLinz, Austria; ^4^Max Planck Institute for Human Cognitive and Brain SciencesLeipzig, Germany

**Keywords:** embodied cognition, episodic memory, virtual reality, memory training, embodied memory

In the last decade, embodiment has dramatically influenced our conception of cognition. In this new frame, episodic memory, and particularly memory decline have been reinterpreted. Interventions supporting memory in the aging population address the connection between mind and body. Here, we discuss the use of Virtual Reality (VR) as an innovative tool to support episodic memory in older adults.

## The embodied nature of episodic memory and its implications for aging

Traditional cognitive models of human mind describe it as processing abstract symbols and following predefined formal rules (Fodor, 1975, 1983).

An alternative view, the embodied cognition approach, claims however that the mind is inherently embodied: this is to say that perceptual and motor systems influence the way we construct concepts, make inferences, and use language (Barsalou, 2008; Shapiro, 2011). Empirical evidence supports this claim in different cognitive domains: in language comprehension (Hauk et al., 2004; Tettamanti et al., 2005; Repetto et al., 2013, 2015b; Repetto, 2014), second language learning (Macedonia et al., 2011; Repetto et al., 2015a; Macedonia and Repetto, 2016), and also in memory (Pezzulo et al., 2010; van Dam et al., 2013; Downing-Doucet and Guérard, 2014; Bochynska and Laeng, 2015; Lagacé and Guérard, 2015). Specifically, it has been argued that the ability to encode and retrieve information is constrained by the actions we can perform upon it and by the limitations/opportunities provided by our bodies (Glenberg, 1997). The embodied nature of memory appears particularly evident for episodic memories. They consist of past events with reference to the individuals themselves as participants of those events (Tulving, 2001, 2002) and linked to a place and a particular time. According to Tulving (2001), the essence of episodic retrieval is the “autonoetic consciousness,” namely the subjective and conscious experience of mentally reliving a personal past event. This includes “all the attendant visual, kinesthetic, and spatial impressions” (Wilson, 2002 p.633).

It has been argued that the ontogeny of episodic memory is strictly connected with the onset of locomotion during infancy, and related to the maturation of the hippocampus (Glenberg and Hayes, 2016). This brain structure, together with the entorhinal cortex, contains different kinds of cells dedicated to different tasks. Hippocampal place cells code location (Ekstrom et al., 2003). Head-direction cells provide a viewing direction on the retrieved contents (Taube, 1998) and allow the generation of an egocentrically coherent representation in medial parietal areas. Grid cells in the entorhinal cortex support the process of updating a viewpoint in relation to self-motion signals (Boccara et al., 2010). Glenberg and Hayes (2016) specifically proposed that the alignment of hippocampal place cells and grid cells related to environmental cues triggers the encoding of space in which personal events are experienced. Embodied navigation and memory are thus strictly connected (Leutgeb et al., 2005; Buzsáki and Moser, 2013).

Miller et al. (2013) developed an hybrid memory test assessing both spatial and episodic memory for a group of epileptic patients implanted with depth electrodes. The test was implemented in a virtual reality environment. In the encoding phase, patients were asked to drive in a virtual city and to deliver different items to stores; in the retrieval phase, patients were prompted to verbally recall the items delivered during navigation. Miller and colleagues found that place cells that fired at a specific location during virtual navigation were also activated during subsequent recall of the item associated to that location. This indicates that activity was reinstated during retrieval in episodic memory.

The embodied approach of memory also accounts for the decline of episodic memory described during aging (Koen and Yonelinas, 2014). Here, the critical element is the reduction of locomotion observed in older individuals. The decrease of locomotion results in the reduction of the opportunities for the hippocampal place cells and grid cells to be tuned with the environment (Glenberg and Hayes, 2016). It stands to reason that, in order to enhance episodic memory in older adults, trainings procedures should target self-locomotion and navigation tasks. To this extent, Virtual Reality systems (VR) could be employed as training tool.

## Virtual reality as a tool to improve episodic memory in the aging population

Virtual reality allows users to create, explore and interact within environments that are perceived as near to reality. Typically, users entering a VR lab feel as being a part of this world and behave as if they were in the real world (Riva and Mantovani, 2012; Riva et al., 2014). Specifically, even if users do not always move their bodies in the real space, users have the subjective perception of being “in action.” The effect of a virtual action on cognitive processing has been demonstrated by Repetto et al. (2015b) who have found that performing a virtual movement with a limb (i.e., virtual run—performed with the legs/feet) speeds up the comprehension of verbs that describe actions performed with the same limb (to kick, performed with legs/feet). On this base, VR can be used to support episodic memory, in particular in elderly (Morganti et al., 2013).

Three main features that VR implements may have an impact on episodic memory in elderly:

First VR allows to experience from an egocentric point of view. This feature places VR in an intermediate position between mere action observation (such as in a video) and real action execution (Serino et al., 2015), with an important rebound on brain activity (Serino et al., 2014). In a fMRI study, Strafella and Paus (2000) have demonstrated that cortical excitability is modified by the observation of movements performed by others. Futhermore, this modulation can be enhanced if the orientation of the movement is egocentric (Maeda et al., 2002). In a recent experiment, Bergouignan et al. (2014) tested the egocentric point of view in relation to encoding and retrieving real-life events. Participants seating in front of a “professor” had a social interaction with him. They saw a real scene filmed and projected through a head-mounted display (HMD) connected to cameras in different locations. The first camera was located exactly over the participants' head, so that participants looked at the scene from the same perspective as without the HMDs. The second camera was located 2 m in front of the participants and rotated 180° to face them. This experimental manipulation yielded two different subjective frames of reference. In the former, the sense of bodily self was located inside of the physical body (in-body condition). In the latter the sense of bodily self was located outside the physical body (out-of-body condition). To induce the illusion of being in one of these two locations, they received a visuo-tactile stimulation on their chest with a rod, synchronous with the scene projected to HMD. After a week, participants' episodic memory of these life events was assessed. Results revealed a poorer recollection for life events encoded in the out-of-body condition. Furthermore, findings indicated that the retrieval of these life events was associated with activity changes in the hippocampus. Considering these findings, VR allowing an egocentric encoding and retrieval, could be a valid training tool for episodic memory.

Second, VR allows active navigation while the user actively explores the environment by manipulating keyboards, joysticks, or controllers. Within a virtual world, the user can choose directions and get the impression of walking or running simply by regulating the motion speed. Being the self-locomotion the base of embodiment and grounding consequently episodic memory (Glenberg and Hayes, 2016) virtual promenades could compensate for the decrease of the spontaneous motion in elderly. Jebara et al. (2014) investigated the effect of different types of virtual navigation on episodic memory in young and older adults. In a virtual environment designed as a city to be explored from inside a car, participants were asked to retrieve the events occurred with questions on “what,” “where,” and “when.” Participants were assigned to four experimental conditions: (1) in the *passive condition* participants were the passengers, with no possibility to interact with the environment; (2) in the *itinerary control*, participants chose the road but did not drive the car; (3) in the *low navigation control*, participants displayed the car on rails they could control with pedals without choosing the directions; (4) in *the high navigation control*, participants drove as in real life choosing also directions. As expected, the low navigation and the itinerary control conditions enhanced episodic memory in both young and older adults. A study by Sauzéon et al. (2016) report similar findings and suggest that active navigation in episodic memory by means of VR benefits memory. In this study, younger and older adults explored two versions (A and B) of a virtual apartment either actively by choosing directions or passively (through a computer-guided tour of the apartment). The participants were instructed to memorize all the objects located in the different rooms for future recall. Performance was measured with different memory tests. The results underlined that active navigation increased object recognition in both groups, but did not influence other memory tasks (free recall, proactive interference, and semantic clustering). The authors discussed this finding as analogy between active navigation and the *enactment effect* (Engelkamp et al., 1994). Thereafter recall and recognition of items of verbal items are enhanced if participants previously execute semantically related actions to the words. The similarity between active virtual navigation and enactment is given because both procedures selectively impact item-specific processing as recognition, but not relational processing (for example semantic clustering of concepts). Following this perspective, active navigation in VR, can tap into item-specific processing (Pedroli et al., 2015), known to be more defective in older adults compared to relational processing (Dennis et al., 2007). Third, VR provides environmental enrichment by using flexible scenarios. They can in turn be implemented with different degrees of complexity. From bi-dimensional (2D) to tri-dimensional perspectives (3D), the amount of spatial information can increase the degree of enrichment. A beneficial effect of environmental enrichment toward hippocampal function in mice (in terms of neurogenesis and hippocampus-dependent learning and memory tasks) has been reported by Kempermann et al. (1997). Specifically, it has been proven that VR place cells can be activated by means of virtual navigation (Harvey et al., 2009). In humans, Clemenson and Stark (2015) have demonstrated that 3D in virtual environments improves memory in tasks known to be related to hippocampal activity. In their study, the authors trained naïve video gamers for 2 weeks on two different videogames. One was based on simple 2D graphics, and one was based on 3D complex graphics. The control group received no training. Visual and memory performance were tested: visual accuracy and visual processing speed in an enumeration task, memory discrimination between highly similar lure items from repeated items, and a spatial memory score. Participants trained with the 3D videogame outperformed both, the 2D gamers and the control group in the discrimination task and in the spatial memory task, but not in the visual task. Being the discrimination task associated with the activity of the hippocampus (Lacy et al., 2010), authors inferred that 3D enriched VR presumably impacts also the hippocampal activity. Taken together, these findings support the view that episodic memory can be enhanced in older adults by means of enriched 3D scenarios. They can stimulate hippocampal activity which in turn supports episodic memory.

However, the use of VR with elderly might have some limitations *per se*: the first affects the usability of different devices such as HMD, joypad or other controllers (Castilla et al., 2013). Elder persons are reluctant toward technology and not used to handle (Manera et al., 2016). The second limitation has to do with the cognitive load of the task. If the task is too demanding, this can have detrimental effects on the memory performance if attentional resources might split between complex motor tasks and memory task (Jebara et al., 2014).

Despite these caveats, these studies pave the way for a new concept of trainings for episodic memory: proposing VR as an effective tool for active navigation in 3D enriched worlds.

## Author contributions

CR, GR conceived the paper. CR, SS wrote the paper. MM, GR revised the paper.

### Conflict of interest statement

The authors declare that the research was conducted in the absence of any commercial or financial relationships that could be construed as a potential conflict of interest. The reviewer NP and the handling Editor declared their shared affiliation, and the handling Editor states that the process nevertheless met the standards of a fair and objective review.
